# Increased risk of cataract surgery in patients with allergic disease: a population based cohort study

**DOI:** 10.1038/s41598-022-25589-1

**Published:** 2022-12-08

**Authors:** Ji-Sun Paik, Kyungdo Han, Gahee Nam, Sun-Kyoung Park, Ho Sik Hwang, Yoon Hong Chun, Kyung-Sun Na

**Affiliations:** 1 Department of Ophthalmology, Yeouido St. Mary’s Hospital, College of Medicine, The Catholic University of Korea, 10, 63-ro, Yeongdeungpo-gu, Seoul, 07345 Republic of Korea; 2 Department of Statistics and Actuarial Science, Soongsil University, Seoul, 06978 Republic of Korea; 3 Department of Pediatrics, Incheon St. Mary’s Hospital, College of Medicine, The Catholic University of Korea, 56, Dongsu-ro, Bupyeong-gu, Incheon, 21431 Republic of Korea

**Keywords:** Corneal diseases, Asthma, Epidemiology

## Abstract

We investigated the association between cataract and allergic diseases, including atopic dermatitis (AD), allergic rhinitis (AR), and asthma using 2,631,015 subjects’ data from the 2009 National Health Insurance Service-Health Screening Cohort in Korea. Each allergic disease was defined as three or more occasions of diagnosis within 1 year with dedicated ICD-10 codes. The primary endpoint was newly received cataract surgery during the follow-up period. In total, 447,883 subjects had at least one allergic disease. During the mean follow-up of 7.8 ± 1.7 years, newly developed cataract surgery was observed in 301,693 subjects (allergic group, n = 69,321; non-allergic group, n = 232,372). After adjusting for demographic characteristics (age, sex), systemic and ocular comorbidities, socioeconomic status, and lifestyle factors (smoking, drinking, regular exercise), the allergic group had a higher hazard ratio (HR) for cataract development compared with the non-allergic group. We further performed a subgroup analysis for patients regarding sex and age. In the subgroup analysis of subjects with AD, men aged < 50 years had a higher HR compared to women of the same age group. In conclusion, subjects with allergic diseases had a higher risk of cataract surgery than their counterparts, and the combination of AD and AR resulted in the highest risk. Particularly, the association was more evident in male than female patients with AD aged < 50 years.

## Introduction

Allergic diseases are common in adults and children, and their prevalence has consistently increased over the last few decades^1^. Atopic dermatitis (AD), allergic rhinitis (AR), and asthma, which comprise the atopic triad, are the most common chronic allergic diseases. They have a common etiology, involving type II immune responses secondary to genetic and allergic factors. Each condition is distinct, with its own phenotype and pathogenesis^1^.

Approximately 25–40% of adults with AD also have ophthalmic problems, including blepharitis, conjunctivitis, tear film disturbances, keratoconus, uveitis, cataract, and retinal detachment^2^. Among these, the risk of developing cataract remains controversial; eye rubbing, inflammation, serum immunoglobulin E level, and glucocorticoid use may or may not be related to cataract. Previous studies have suggested that among the allergic diseases, AD is most frequently associated with an increased incidence of cataract^3^. In particular, shield-shaped anterior and posterior subcapsular cataracts are characteristic ocular comorbidities associated with AD^4^.

Most of the available studies that explored the association between cataracts and asthma, or AR have been limited because of the repetitive use of systemic corticosteroids and long-term intranasal corticosteroids among the study populations. Furthermore, reports that investigated cataracts of all severities, including mild to moderate disease, are scarce^5–7^. Interestingly, even in the same cohort population, studies that investigated the association between allergic diseases and the incidence of cataracts have shown different results. Lee et al. studied a cohort of adults with allergic diseases and concluded that the incidences of cataracts and glaucoma were significantly associated with AD^8^. However, another study of the same cohort reported that the incidence of cataracts was significantly associated with asthma and AR, but not with AD^9^. These differences may be because of variations in the disease definitions and study methods. The present study aimed to evaluate the association between allergic diseases and the risk of cataract in Koreans using the National Health Insurance Service-National Sample Cohort (NHIS-NSC) as the database. To eliminate selection bias, the study population was restricted to only visually affected patients with cataract who underwent cataract surgery. Additionally, the study population was categorized into women and men aged < 50 years and ≥ 50 years to further adjust the sex and age effect. The effect of pre-existing allergic disease on the incidence of cataracts was investigated.

## Results

### Baseline characteristics of the study population

Table 1 shows the baseline characteristics according to the presence of cataracts and allergic diseases, such as any allergy, AD, asthma, and AR. Of the total 2,631,015 study subjects, 301,693 (11.4%) underwent cataract surgery at least in one eye. Furthermore, 447,883 (17.0%), 10,631 (0.40%), 91,673 (3.5%), and 400,037 (16.7%) subjects had allergy, AD, asthma, and AR, respectively. The subjects in the cataract and allergic disease groups were older than those in the control group, and the former group had a significantly lower proportion of men than the latter group. The proportions of heavy alcohol consumers, current smokers, and individuals who did not exercise regularly were significantly lower in the cataract and allergic disease groups, excluding the asthma group, than in the control group. Cardiovascular risk factors, such as hypertension, diabetes mellitus (DM), and dyslipidemia, and obesity parameters, including body mass index (BMI) and waist circumference, were higher in the cataract and allergic disease groups than in the control group.

**Table 1:** General characteristics of study population.

	Cataract		P value	Any allergy		P value	Atopic dermatitis		P value	Asthma		P value	Rhinitis		P value
	No	Yes		No	Yes		No	Yes		No	Yes		No	Yes	
Number	2,329,322	301,693		2,183,132	447,883		2,620,384	10,631		2,539,342	91,673		2,330,978	400,037	
Sex	1,203,440 (51.66)	131,491 (43.58)	< 0.0001	1,147,273 (52.55)	187,658 (41.9)	< 0.0001	1,329,867 (50.75)	5064 (47.63)	< 0.0001	1,297,738 (51.11)	37,193 (40.57)	< 0.0001	1,168,195 (52.36)	166,736 (41.68)	< 0.0001
Age	52.4 ± 9.48	63.09 ± 9.15	< 0.0001	53.3 ± 9.91	55.22 ± 10.52	< 0.0001	53.62 ± 10.03	56.45 ± 10.66	< 0.0001	53.43 ± 9.94	59.24 ± 10.99	< 0.0001	53.45 ± 9.98	54.62 ± 10.29	< 0.0001
WC	80.92 ± 8.58	82.77 ± 8.36	< 0.0001	81.1 ± 8.55	81.29 ± 8.68	< 0.0001	81.13 ± 8.57	81.93 ± 8.85	< 0.0001	81.08 ± 8.55	82.52 ± 8.9	< 0.0001	81.14 ± 8.56	81.11 ± 8.63	0.0214
BMI	23.93 ± 3.02	24.24 ± 3.06	< 0.0001	23.94 ± 3.02	24.09 ± 3.07	< 0.0001	23.97 ± 3.03	24.12 ± 3.1	< 0.0001	23.96 ± 3.02	24.31 ± 3.32	< 0.0001	23.95 ± 3.03	24.07 ± 3.03	< 0.0001
**Smoking**			< 0.0001			< 0.0001			< 0.0001			< 0.0001			< 0.0001
Non	1,445,317 (62.05)	211,589 (70.13)		1,343,351 (61.53)	313,555 (70.01)		1,649,986 (62.97)	6,920 (65.09)		1,592,541 (62.71)	64,365 (70.21)		1,375,810 (61.67)	281,096 (70.27)	
Past	368,002 (15.8)	44,390 (14.71)		344,539 (15.78)	67,853 (15.15)		410,638 (15.67)	1,754 (16.5)		399,227 (15.72)	13,165 (14.36)		351,498 (15.76)	60,894 (15.22)	
Current	516,003 (22.15)	45,714 (15.15)		495,242 (22.68)	66,475 (14.84)		559,760 (21.36)	1,957 (18.41)		547,574 (21.56)	14,143 (15.43)		503,670 (22.58)	58,047 (14.51)	
**Drinking**			< 0.0001			< 0.0001			< 0.0001			< 0.0001			< 0.0001
Non	1,313,135 (56.37)	206,555 (68.47)		1,227,556 (56.23)	292,134 (65.23)		1,512,800 (57.73)	6,890 (64.81)		1,455,081 (57.3)	64,609 (70.48)		1,260,722 (56.51)	258,968 (64.74)	
Mild	838,809 (36.01)	77,209 (25.59)		782,840 (35.86)	133,178 (29.73)		912,859 (34.84)	3159 (29.71)		893,200 (35.17)	22,818 (24.89)		794,964 (35.63)	121,054 (30.26)	
Heavy	177,378 (7.62)	17,929 (5.94)		172,736 (7.91)	22,571 (5.04)		194,725 (7.43)	582 (5.47)		191,061 (7.52)	4246 (4.63)		175,292 (7.86)	20,015 (5)	
Exercise	468,486 (20.11)	62,812 (20.82)	< 0.0001	438,083 (20.07)	93,215 (20.81)	< 0.0001	529,055 (20.19)	2243 (21.1)	0.0199	513,458 (20.22)	17,840 (19.46)	< 0.0001	447,059 (20.04)	84,239 (21.06)	< 0.0001
Income*	504,299 (21.65)	66,144 (21.92)	0.0006	470,628 (21.56)	99,815 (22.29)	< 0.0001	567,988 (21.68)	2455 (23.09)	0.0004	549,585 (21.64)	20,858 (22.75)	< 0.0001	481,297 (21.57)	89,146 (22.28)	< 0.0001
DM	232,873 (10)	59,537 (19.73)	< 0.0001	238,649 (10.93)	53,761 (12)	< 0.0001	290,879 (11.1)	1531 (14.4)	< 0.0001	278,898 (10.98)	13,512 (14.74)	< 0.0001	246,127 (11.03)	46,283 (11.57)	< 0.0001
HTN	740,043 (31.77)	159,099 (52.74)	< 0.0001	726,172 (33.26)	172,970 (38.62)	< 0.0001	894,634 (34.14)	4508 (42.4)	< 0.0001	855,688 (33.7)	43,454 (47.4)	< 0.0001	749,637 (33.6)	149,505 (37.37)	< 0.0001
Dyslipidemia	495,816 (21.29)	95,423 (31.63)	< 0.0001	473,775 (21.7)	117,464 (26.23)	< 0.0001	588,109 (22.44)	3130 (29.44)	< 0.0001	564,175 (22.22)	27,064 (29.52)	< 0.0001	487,761 (21.86)	103,478 (25.87)	< 0.0001
CKD	164,461 (7.06)	38,810 (12.86)	< 0.0001	163,737 (7.5)	39,534 (8.83)	< 0.0001	202,235 (7.72)	1036 (9.75)	< 0.0001	193,251 (7.61)	10,020 (10.93)	< 0.0001	169,213 (7.58)	34,058 (8.51)	< 0.0001
Glaucoma	26,386 (1.13)	10,349 (3.43)	< 0.0001	26,661 (1.22)	10,074 (2.25)	< 0.0001	36,425 (1.39)	310 (2.92)	< 0.0001	34,315 (1.35)	2,420 (2.64)	< 0.0001	27,811 (1.25)	8924 (2.23)	< 0.0001
RVO	1366 (0.06)	681 (0.23)	< 0.0001	1576 (0.07)	471 (0.11)	< 0.0001	2037 (0.08)	10 (0.09)	0.5468	1923 (0.08)	124 (0.14)	< 0.0001	1638 (0.07)	409 (0.1)	< 0.0001

WC, waist circumstance; BMI, body mass index; DM, diabetes mellitus; HTN, hypertension; CKD, chronic kidney disease; RVO, retinal vascular occlusion.

*Low income indicates the low quadrant of income level (25%).

### Incidence and risk of cataract in patients with allergic diseases

**Figure 1:**
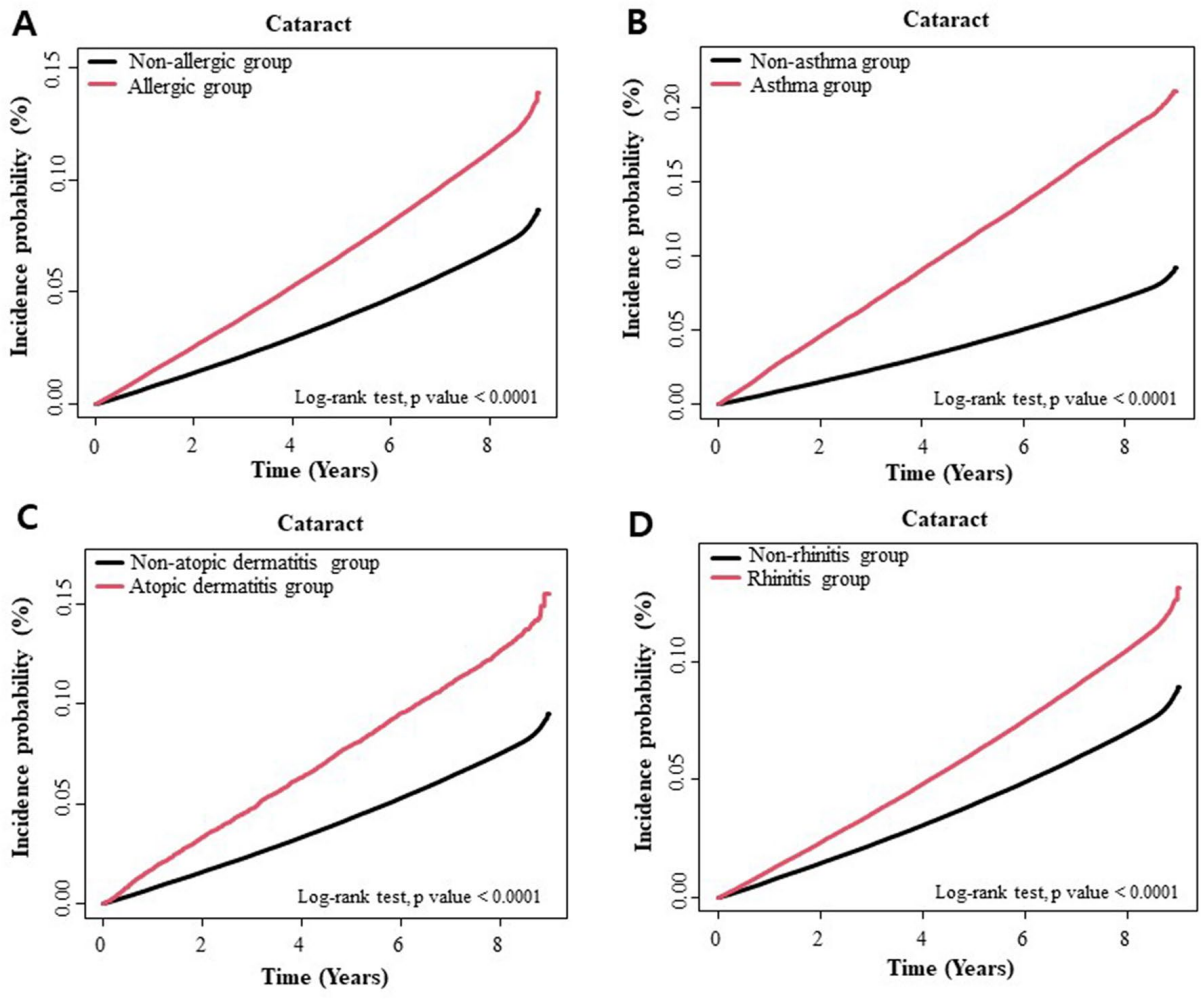
Comparison of cumulative incidence probability of cataract according to the allergic disease (**a**), asthma (**b**), atopic dermatitis (**c**), and allergic rhinitis (**d**). The incidence probability of cataract increases more over time in the allergic group than in non-allergic group for all different kinds of allergic disease.

During a mean follow-up of 7.8 ± 1.7 years, newly developed cataract was observed in 301,693 subjects. Of these, 69,321 (19.74/1000 person-years) and 232,372 (13.64/1000 person-years) subjects were from the allergic and non-allergic groups, respectively. After adjusting for confounding factors (age, sex, smoking, drinking, regular exercise, low income, DM, hypertension, dyslipidemia, chronic kidney disease [CKD], BMI, glaucoma, and retinal vascular occlusion [RVO]), the allergic group had a higher hazard ratio (HR) of cataract development than the non-allergic group (HR = 1.241; 95% confidence interval [CI], 1.230–1.252) (Table 2). Among one, two, or three allergic diseases, three allergic diseases had shown the highest HR of cataract development (HR = 1.409; 95% CI, 1.213–1.637). Figure 1 shows a comparison of the cumulative incidence probabilities of cataracts according to any allergy, asthma, AD, and rhinitis. The incidence probability of cataract was higher over time in the allergic group than in the non-allergic group for all types of allergic diseases.

**Table 2:** Risk for cataract development according to presence of allergic diseases.

Subgroups	Total n	Cataract	Duration	Rate	Model 1	Model 2	Model 3	Model 4	Model 5
					HR (95% CI)	HR (95% CI)	HR (95% CI)	HR (95% CI)	HR (95% CI)
Non-allergic group	2,183,132	232,372	17,043,785.47	13.6339	1 (Ref.)	1 (Ref.)	1 (Ref.)	1 (Ref.)	1 (Ref.)
Allergic group	447,883	69,321	4,019,777.21	19.7421	1.473 (1.463, 1.490)	1.272 (1.251, 1.291)	1.272 (1.251, 1.292)	1.243 (1.223, 1.260)	1.241 (1.230, 1.252)
**One allergic disease**	394,215	58,407	3,004,119.29	19.4423	1.429 (1.416, 1.442)	1.238 (1.227, 1.249)	1.238 (1.227, 1.249)	1.218 (1.207, 1.229)	1.211 (1.200, 1.222)
Atopic dermatitis (AD)	6,384	1115	47,448.2	23.4993	1.731 (1.632, 1.836)	1.373 (1.295, 1.457)	1.374 (1.296, 1.458)	1.339 (1.263, 1.42)	1.328 (1.252, 1.409)
Asthma	41,158	9106	287,687.34	31.6524	2.347 (2.298, 2.397)	1.230 (1.204, 1.256)	1.230 (1.204, 1.256)	1.200 (1.175, 1.225)	1.195 (1.170, 1.220)
Allergic rhinitis (AR)	346,673	48,186	2,668,983.75	18.0541	1.326 (1.313, 1.339)	1.237 (1.225, 1.249)	1.237 (1.225, 1.249)	1.219 (1.207, 1.231)	1.212 (1.200, 1.224)
**Two allergic diseases**	52,878	10,742	384,683.16	27.9243	2.062 (2.023, 2.102)	1.403 (1.376, 1.430)	1.403 (1.376, 1.430)	1.362 (1.335,1.388)	1.347 (1.321,1.374)
AD plus asthma	304	80	2098.81	38.1168	2.860 (2.300, 3.557)	1.387 (1.116, 1.724)	1.383 (1.112,1.718)	1.321 (1.061, 1.645)	1.302 (1.046, 1.621)
AD plus AR	3153	590	23,319.09	25.3012	1.865 (1.72, 2.022)	1.487 (1.372, 1.613)	1.487 (1.372, 1.612)	1.449 (1.336, 1.570)	1.436 (1.324, 1.557)
Asthma plus AR	49,421	10,072	359,265.26	28.035	2.070 (2.029, 2.112)	1.398 (1.370, 1.426)	1.398 (1.370, 1.426)	1.357 (1.330, 1.384)	1.343 (1.316, 1.370)
**Three allergic diseases**	790	171	5610.82	30.6551	2.259 (1.944, 2.624)	1.506 (1.296, 1.750)	1.504 (1.294, 1.747)	1.424 (1.226, 1.655)	1.409 (1.213, 1.637)

CI, confidence interval; HR, hazard ratio.

Model 1 non-adjusted. Model 2 is adjusted for age, sex. Model 3 is adjusted for age, sex, smoke, drink, regular exercise, and low income. Model 4 is adjusted for age, sex, smoking, drinking, regular exercise, low income, diabetes mellitus (DM), hypertension, dyslipidemia, chronic kidney disease (CKD), and body mass index (BMI). Model 5 is adjusted for age, sex, smoking, drinking, regular exercise, low income, DM, hypertension, dyslipidemia, CKD, BMI, glaucoma, and retinal vascular occlusion.

### Subgroup analysis of the association between cataract and atopic dermatitis

In all subgroups, subjects with any allergy, AD, AR, and asthma had an increased risk for cataract development (Tables S1–3, Table 3). In the subjects with allergy, there were significant differences in the risk of cataract development in the subgroups, except for hypertension, CKD, and regular exercise (Table S1). Interestingly, in subjects with AD, there were sex differences in the age and sex subgroups. After adjusting for confounding factors (age, sex, smoking, drinking, regular exercise, low income, DM, hypertension, dyslipidemia, CKD, and BMI), the male group aged < 50 years showed higher HR (1.713) (95% CI, 1.338–2.194) than the female group aged ≥ 50 years (HR, 1.193; 95% CI, 1.119–1.273) (Table 3). These trends were not observed in the subgroup analyses of other allergic diseases, such as any allergy, asthma, and AR (Tables S1–3).

**Table 3:** Association between cataract and atopic dermatitis subjects according to subgroup.

Subgroup	Atopic dermatitis	N	Cataract	Follow-up duration^a^	Incidence rate^b^	Model 4	P for interaction
**Sex, Age**							
Male, Age < 50	No	563,563	15,006	4,613,370.9	3.2527	1 (Ref.)	0.0002
	Yes	1360	63	11,048.04	5.7024	1.713 (1.338, 2.194)	
Male, Age ≥ 50	No	766,304	115,523	5,713,785.56	20.2183	1 (Ref.)	
	Yes	3704	899	25,569.97	35.1584	1.42 (1.33, 1.516)	
Female, Age < 50	No	491,169	11,575	4,053,559.5	2.8555	1 (Ref.)	
	Yes	1825	60	15,023.8	3.9937	1.326 (1.029, 1.709)	
Female, Age ≥ 50	No	799,348	157,633	5,979,005.85	26.3644	1 (Ref.)	
	Yes	3742	934	26,835.11	34.8051	1.193 (1.119, 1.273)	
**BMI**							
< 25	No	1,705,961	183,985	13,270,403.13	13.8643	1 (Ref.)	0.9586
	Yes	6665	1128	49,392.04	22.8377	1.305 (1.231, 1.384)	
≥ 25	No	914,423	115,752	7,089,318.69	16.3277	1 (Ref.)	
	Yes	3966	828	29,084.88	28.4684	1.305 (1.218, 1.397)	
**DM**							
No	No	2,329,505	240,647	18,265,187.96	13.1752	1 (Ref.)	0.3724
	Yes	9100	1509	68,126.72	22.1499	1.307 (1.243, 1.375)	
Yes	No	290,879	59,090	2,094,533.86	28.2115	1 (Ref.)	
	Yes	1531	447	10,350.21	43.1875	1.293 (1.178, 1.419)	
**Hypertension**							
No	No	1,725,750	141,796	13,735,823.39	10.3231	1 (Ref.)	0.7057
	Yes	6123	798	47,284.35	16.8766	1.287 (1.201, 1.38)	
Yes	No	894,634	157,941	6,623,898.42	23.8441	1 (Ref.)	
	Yes	4508	1158	31,192.58	37.1242	1.315 (1.241, 1.394)	
**Dyslipidemia**							
No	No	2,032,275	205,049	15,907,194.72	12.8903	1 (Ref.)	0.1425
	Yes	7501	1221	56,085.67	21.7703	1.334 (1.261, 1.411)	
Yes	No	588,109	94,688	4,452,527.1	21.2661	1 (Ref.)	
	Yes	3130	735	22,391.25	32.8253	1.266 (1.177, 1.361)	
**Smoking**							
Non	No	1,649,986	210,270	12,794,735.34	16.4341	1 (Ref.)	0.2778
	Yes	6920	1319	51,249.64	25.7368	1.274 (1.207, 1.345)	
Past	No	410,638	44,062	3,189,597.86	13.8143	1 (Ref.)	
	Yes	1754	328	12,734.01	25.7578	1.381 (1.239, 1.539)	
Current	No	559,760	45,405	4,375,388.62	10.3774	1 (Ref.)	
	Yes	1957	309	14,493.28	21.3202	1.366 (1.221, 1.528)	
**CKD**							
No	No	2,418,149	261,225	18,892,311.73	13.8271	1 (Ref.)	0.4444
	Yes	9595	1658	71,554.98	23.171	1.309 (1.247, 1.374)	
Yes	No	202,235	38,512	1,467,410.09	26.2449	1 (Ref.)	
	Yes	1036	298	6921.95	43.0514	1.29 (1.151, 1.446)	
**Drinking**							
Non	No	1,512,800	205,134	11,610,040.98	17.6687	1 (Ref.)	0.8412
	Yes	6890	1421	49,955.5	28.4453	1.309 (1.243, 1.379)	
Mild	No	912,859	76,767	7,230,603.14	10.617	1 (Ref.)	
	Yes	3159	442	24,238.68	18.2353	1.275 (1.161, 1.4)	
Heavy	No	194,725	17,836	1,519,077.7	11.7413	1 (Ref.)	
	Yes	582	93	4282.74	21.7151	1.368 (1.116, 1.677)	
**Exercise**							
No	No	2,091,329	237,383	16,237,317.47	14.6196	1 (Ref.)	0.1304
	Yes	8388	1498	61,975.96	24.1707	1.284 (1.22, 1.351)	
Yes	No	529,055	62,354	4,122,404.35	15.1256	1 (Ref.)	
	Yes	2,243	458	16,500.96	27.756	1.378 (1.257, 1.511)	
**Low income**							
No	No	2,052,396	234,049	15,949,654.08	14.6742	1 (Ref.)	0.3703
	Yes	8176	1500	60,374.39	24.845	1.293 (1.229, 1.36)	
Yes	No	567,988	65,688	4,410,067.74	14.895	1 (Ref.)	
	Yes	2455	456	18,102.54	25.1898	1.349 (1.231, 1.48)	
**RVO**							
No	No	2,618,347	299,059	20,346,268.44	14.6985	1 (Ref.)	0.962
	Yes	10,621	1953	78,419.05	24.9047	1.304 (1.248, 1.364)	
Yes	No	2037	678	13,453.38	50.3962	1 (Ref.)	
	Yes	10	3	57.88	51.8319	1.249 (0.4,3.901)	
**Glaucoma**							
No	No	2,583,959	289,497	20,109,020.93	14.3964	1 (Ref.)	0.4331
	Yes	10,321	1847	76,524.57	24.136	1.31 (1.251, 1.371)	
Yes	No	36,425	10,240	250,700.89	40.8455	1 (Ref.)	
	Yes	310	109	1952.36	55.83	1.233 (1.021, 1.489)	

Model 4 is adjusted for age, sex, smoking, drinking, regular exercise, low income, DM, hypertension, dyslipidemia, CKD, and BMI.

BMI, body mass index; DM, diabetes mellitus; CKD, chronic kidney disease; RVO, retinal vascular occlusion.

^a^Follow-up duration is in person-years.

^b^Incidence rate is presented as 1000 person-years.

## Discussion

The current study was a nationwide, population-based cohort study investigating the risk of cataract surgery in patients with allergic diseases. We found that the subjects with allergic diseases were at an increased risk of visually affected cataracts, which would lead to cataract surgery. Among these, young male subjects were more sensitive to the association between allergic diseases and cataract surgery incidence than their counterparts. In addition, the combination of AD and AR resulted in the highest risk.

The association between AD and cataract has been suggested as a case report of dermatology study before the 1950s^10–13^. However, whether allergic diseases increase the risk of cataract development remains unclear, or the mechanism between allergic diseases and cataracts remains unknown. Previous population-based studies have reported conflicting results^4,8,9^. A longitudinal cohort study included pediatric subjects aged between 12 and 20 years using claims data and compared the incidence probabilities of cataract development and cataract surgery between AD and propensity score-matched controls^4^. Definitions of AD, cataract development, and cataract surgery were identified using diagnostic codes. The incidence of cataract development and cataract surgery was rare in the AD and control groups (0.12% vs. 0.14% and 0.04% vs. 0.02%, respectively). The results showed that the development of cataracts was not different between the AD and control groups, but cataract surgery was performed more frequently in the AD cohort than in the control group (0.075% vs. 0.041%; 95% CI, 0.017%–0.050%; P = 0.02). The limitation of the study was the possibility of underestimation of cataracts because the subjects were defined based on diagnostic codes. To reduce selection bias, the incidence of cataract surgery was evaluated; however, childhood cataract tends to be neglected because of lower awareness of vision discomfort, and the decision to perform cataract surgery is postponed because of the need for general anesthesia. We enrolled a different age group of over 20 years and found that subjects aged < 50 years tended to have an increased risk of cataract surgery when diagnosed and treated with AD, AR, and asthma in the subgroup analysis. We classified the subjects based on age because aging has been shown to be the strongest risk factor for the development of cataract^14^. Thus, the analysis of other factors that contribute to an increased prevalence of cataracts might have been masked by the age of the subjects in the current study.

The following two cross-sectional population-based studies using the same database obtained from the Fifth Korean National Health and Nutrition Examination Survey (KNHANES-V, 2010–2012) showed different results^8,9^. Lee et al.^9^ investigated the association between cataract development and allergic diseases in a population aged > 19 years. Cataract was defined as a history of ophthalmology or cataract surgery, and allergic diseases were defined as “yes” responses to the questions of diagnostic history. After adjusting the confounding factors, the odds ratio (OR) for cataract according to the presence of allergic diseases revealed that AR and asthma were significantly associated with cataracts (OR = 1.511, 95% CI = 1.120–2.039 and OR = 1.565, 95% CI = 1.192–2.054, respectively), whereas AD was not associated with cataract development. However, another nationwide study using the same database of over the age of 20 years reported different results^8^. Cataract was defined according to the standard Lens Opacities Classification System III. Through comparison with standard photographs, cataracts were categorized as cortical, nuclear, posterior subcapsular, or mixed. Subjects with AD were defined as those who answered “yes” to the question of a history of AD diagnosis by physicians. After adjusting the confounding factors, cataract was significantly associated with AD (OR, 1.85; 90% CI, 1.06–3.22; P = 0.031), and the patients with AD had a higher prevalence of ophthalmic surgery compared to those without AD (OR, 2.22; 95% CI, 1.07–4.61; P = 0.032).

A possible explanation for these conflicting results may be the misclassification of patients with cataract. Several clinical classifications and measurements have been used to evaluate cataracts^15^. However, these examinations have a common limitation of the subjective nature of grading and are influenced by observers with various slit lamp settings and training levels^16,17^. This could lead to inconsistencies of cataract diagnosis and interobserver bias. Moreover, recall bias in which the AD subjects were classified as the AD group by survey is a common limitation of studies using the KHANES database. In this study, we identified all subjects with AD, AR, and asthma using the relevant diagnostic code with repeated hospital visits more than three times a year and included only those subjects who underwent cataract surgery with a wash-up period of 1 year of cataract diagnosis or surgery prior to enrollment of cohort observation to reduce selection bias.

Although the pathogenesis of cataract in allergic diseases has been elucidated, several mechanisms have been postulated to explain this association. First, allergic diseases and cataracts share similar risk factors. Allergic diseases are related to individual components of metabolic risk, such as elevated blood pressure, blood glucose, and high cholesterol levels^18,19^, which may partly explain the increased incidence of cataract formation^20,21^. Other factors, including inflammation, clinical severity of AD, oxidative stress, and corticosteroid use, may contribute to cataract formation. A previous study showed that higher levels of aqueous flare were reported in atopic cataract and suggested the possible role of the blood-aqueous barrier in cataract formation in patients with AD^22^. In addition, tissue-destroying major basic protein derived from eosinophils was found in atopic cataracts, but not in any of the senile cataracts^23^. However, a previous study failed to reveal an increased risk of cataract development based on the severity of allergic diseases or systemic skin lesions. Instead, cataract development was observed in some patients with only facial involvement^24^. Similarly, we found that subjects with AD showed higher HR for cataract surgery compared to those with AR and asthma, indicating that eye rubbing or facial skin involvement may be an additive risk factor in cataract development. A previous prospective study of patients with AD showed that subjects with facial AD, contact lenses, or both may rub their eyes more frequently than those with lesions on other body parts, increasing their risk of cataract progression^25^.

In the subgroup analyses, this study found that AD, AR, and asthma were significantly associated with an increased incidence of cataracts, with a higher HR in men aged < 50 years. This result is unexpected, as previous studies have found that AD and respiratory allergy predominantly affect women after adolescence, following the start of menarche^26,27^. It was hypothesized that estrogen was responsible for the higher incidence and severity of allergic diseases in women after menarche^27^. Factors other than typical allergic inflammatory responses may contribute to the pathogenesis and underlying mechanism related to the higher incidence of cataracts in patients aged < 50 years. In a previous prospective study of presenile nuclear cataracts in individuals aged 30–49 years, 74 of the 266 patients had presenile nuclear cataracts. Of these, 63.5% were male. Current smoking habits, non-exercise or high amount of physical exercise, asthma, tuberculosis, and iron deficiency status were significantly associated with disease incidence^28^. Our results concurred with the finding that respiratory allergy was associated with an early incidence of cataracts in men. Another explanation for sex differences in the association between allergic diseases and cataract surgery is smoking. Various studies have shown that smoking is a risk factor for early-onset cataract due to oxidative stress and triggers the worsening of symptoms by increasing the number of inflammatory cytokines in allergic diseases^27,29,30^. Alternatively, other studies have hypothesized that the higher incidence of cataracts in men aged < 50 years may be associated with the disease control status of young men. We used the National Health Insurance Database and analyzed the medical service utilization of patients with AD, AR, and asthma over 5 years. We found that inpatient admissions and outpatient visits were predominant in women in all three disease groups. In contrast, there was a male predominance in emergency visits in all three disease groups^31^. This result suggests that failure to control the disease and acute exacerbations occurred more commonly in men than in women. The higher incidence of cataracts in men aged < 50 years may be associated with indifference and/or poor adherence to allergic disease management among young men. Another explanation may be that men aged < 50 years may have a greater tendency to undergo early surgical intervention than women because men are more socially active than women in Korea^32^.

This study has several strengths, including a large cohort of men and women, stable multiple-year follow-up, and high participation rate. However, this study has some limitations that should be discussed. First, the nature of the study design, using a claims database, could lead to Berkson’s bias. As the operation definition using the ICD-10 diagnostic code was included in the claims data, misclassification bias and the possibility of underreporting or overreporting cannot be excluded. To increase diagnostic accuracy, we defined AD, AR, and asthma as having at least three records of diagnoses per year in the current study^33^. Frequent visits to hospitals could help to enroll a higher number of subjects with active disease conditions. Second, because this was a nationwide study conducted in a single country with single race, the results may not be generalizable to all populations. This may be the strength of the study to some extent because it prevents the confounding effect of racial, cultural, environmental, and climate differences. As Koreans have relatively uniform genetic and environmental influences, the results may be more consistent than those of other large, population-based studies. Third, the history of steroid treatment for ocular allergy was not considered in this claims data analysis. Thus, it is difficult to evaluate the effect of topical and systemic steroid therapy on the development of cataracts using our claims data. The route (oral, skin, eye, and inhaled), dosage, and strength of steroid medications are highly variable, which hinders accurate measurement of the total amount of systemic steroids. We cautiously assumed that patients with the allergic triad tend to be treated with systemic and local steroids. Future prospective clinical trials comparing the incidence of cataract between patients receiving steroid and non-steroid therapy for allergy may be needed, although ethical issues should be solved. Otherwise, concurrent use of claims data and individual chart review may suggest a possible relationship between steroid therapy and cataract in patients with allergy, because certain types of cataract (i.e., posterior subcapular cataract) are known to be induced by drugs. Finally, observational studies using claims data limit causal inference with the possibility of residual confounding.

In conclusion, long-term follow-up data in the national claims database revealed that the allergic diseases increased the incidence of visually threatening cataracts (cataract surgery) in adults. Patients with AD and AR had the highest risk of cataract surgery among all allergic disease combinations. Adult patients with AD, especially middle-aged men, require screening for a combination of other allergic conditions and regular ophthalmologic examination for the future risk of cataract.

## Methods

### Study design and database

This was a population-based cohort study that used data from the NHIS in Korea. The NHIS provides comprehensive medical care coverage to 97% of the Korean population, including a standardized national health screening program for registrants aged 20 years or older every 2 years. It also provides data on demographics, socioeconomic status, medical treatments, procedures, and disease diagnoses according to the International Classification of Disease, Tenth Revision (ICD-10).

All subjects in the NHIS database are recommended to undergo national health checkups every 2 years. The health checkups include anthropometric data, blood pressure, and laboratory data, such as fasting glucose, cholesterol, and serum creatinine levels. Information on the previous medical history and lifestyle factors, including smoking, alcohol consumption, and regular physical activity, was collected using self-reported questionnaires. All methods were performed in accordance with relevant guidelines and regulations. This study complies with the principles of the Declaration of Helsinki. The Institutional Review Board of the Catholic University of Korea approved this study, and the need for informed consent for study was waived as part of the study approval.

### Establishment of study cohort

We initially selected 4,234,339 subjects aged older than 20 years who had undergone the NHIS health checkup from January 1, 2009, to December 31, 2009. In total, 1,338,019 subjects aged younger than 40 years and 114, 292 subjects with missing data were excluded. Moreover, 115,094 subjects with previous cataract surgery and 35,919 subjects diagnosed with cataract within 1 year from January 1, 2009, were also excluded. A total of 2,631,015 subjects were finally selected and observed through December 31, 2018. Considering the practice pattern for allergic diseases in Korea, prevalent allergic disease was defined as three or more occasions of diagnosis within 1 year with dedicated ICD-10 codes registered by physicians at the outpatient clinic.

### Primary outcome and follow-up

The primary endpoint of this study was newly received cataract surgery at least once during the follow-up period. The cataract surgery group consisted of all subjects with a Korean Electronic Data Interchange (KEDI) code for cataract surgery. For each patient, cataract surgery was defined as the simultaneous claim of either “extracapsular or intracapsular” extraction (KEDI code S5111) or “phacoemulsification” (KEDI code S5119) and “primary intraocular lens implantation” (KEDI code S5117) on the same day. Procedures combined with vitrectomy or glaucoma surgery were excluded. The cohort was observed until the occurrence of the primary endpoint, cataract surgery, emigration, or until the end of the study period, whichever came first.

### Definition of allergic diseases and comorbidities

The records of the ICD-10 Clinical Modification codes were used to define allergic diseases. The atopic triad, including asthma (J45-46), AR (J301-304), and AD (L20), was used to define allergic diseases. Subjects with at least one atopic disease were classified into the allergic disease group. The non-allergic group consisted of subjects without the atopic triad. To improve accuracy, data on allergic diseases were refined by dividing subjects according to the atopic triad diagnosis and number of clinic visits (three or more times per year) to diagnose each disease, which was validated in a previous epidemiological study in Korea^33^. Comorbidities, including hypertension (I10–I15), diabetes mellitus (E11–E14), and dyslipidemia (E78) were also defined on the basis of ICD-10 codes, as described in previous studies^33^.

### Statistical analyses

Categorical variables are presented as numbers and relative frequencies (percentages) and were compared using the χ^2^ test. Normally distributed continuous variables are expressed as mean ± standard deviation and were analyzed using the independent t-test or analysis of variance test. The incidence rate of cataracts was calculated by dividing the number of incident cases by the total follow-up duration, which is presented as events per 1000 person-years. The HR and 95% CI for cataract development were analyzed using the Cox proportional hazards model. The following multivariate adjusted Cox proportional hazards models were applied: model 1 was non-adjusted; model 2 was adjusted for age (continuous variables) and sex; model 3 was adjusted for smoking, drinking, exercise, and income in addition to model 2; model 4 was adjusted for DM, hypertension, dyslipidemia, CKD, and BMI in addition to model 3; and model 5 was adjusted for glaucoma and RVO in addition to model 4.

Additionally, subgroup analyses were performed according to the following confounding factors: age, sex, BMI, DM, hypertension, dyslipidemia, smoking, CKD, drinking, exercise, low income, sex and age, RVO, and glaucoma. The Bonferroni correction was used for multiple comparisons in the subgroups. The cataract-free survival curve was estimated using the log-rank test. Statistical significance was defined as two-sided P < 0.05. All statistical analyses were performed using SAS (version 9.4, SAS Institute Inc., Cary, NC, USA).

## Supplementary Information

Supplementary Tables.

## Data Availability

The datasets generated during and/or analyzed during the present study are available from the corresponding author on reasonable request.
