# Supplementary material for: Increased risk of cataract surgery in patients with allergic disease: a population based cohort study
Source: Sci Rep. 2022 Dec 8;12:21258. doi: 10.1038/s41598-022-25589-1 (PMC9732285; doi:10.1038/s41598-022-25589-1)
Supplement: Supplementary file 1 — Supplementary Tables. [file 41598_2022_25589_MOESM1_ESM.docx]

eTable 1. Association between cataract and allergic subjects according to subgroup.

| **Subgroup** | **Allergy** | **N** | **Cataract** | **Follow-up duration*** | **Incidence rate†** | **Model 4** | **P for interaction** |
| --- | --- | --- | --- | --- | --- | --- | --- |
| **Sex, Age** |  |  |  |  |  |  |  |
| **Male, Age < 50** | **No** | 500,574 | 13,082 | 4,097,214.09 | 3.1929 | 1(Ref.) | <.0001 |
|  | **Yes** | 64,349 | 1,987 | 527,204.85 | 3.7689 | 1.2(1.144,1.258) |  |
| **Male, Age ≥ 50** | **No** | 646,699 | 91,343 | 4,858,569.93 | 18.8004 | 1(Ref.) |  |
|  | **Yes** | 123,309 | 25,079 | 880,785.61 | 28.4734 | 1.261(1.243,1.279) |  |
| **Female, Age < 50** | **No** | 403,601 | 9,211 | 3,331,203.13 | 2.7651 | 1(Ref.) |  |
|  | **Yes** | 89,393 | 2,424 | 737,380.17 | 3.2873 | 1.181(1.129,1.235) |  |
| **Female, Age ≥ 50** | **No** | 632,258 | 118,737 | 4,756,798.32 | 24.9615 | 1(Ref.) |  |
|  | **Yes** | 170,832 | 39,830 | 1,249,042.64 | 31.8884 | 1.214(1.2,1.228) |  |
| **Sex** |  |  |  |  |  |  |  |
| **Male** | **No** | 1,147,273 | 104,425 | 8,955,784.02 | 11.6601 | 1(Ref.) | 0.0025 |
|  | **Yes** | 187,658 | 27,066 | 1,407,990.46 | 19.2231 | 1.252(1.235,1.269) |  |
| **Female** | **No** | 1,035,859 | 127,948 | 8,088,001.46 | 15.8195 | 1(Ref.) |  |
|  | **Yes** | 260,225 | 42,254 | 1,986,422.81 | 21.2714 | 1.217(1.203,1.23) |  |
| **BMI** |  |  |  |  |  |  |  |
| **< 25** | **No** | 1,427,295 | 143,917 | 11,152,542.44 | 12.9044 | 1(Ref.) | 0.0488 |
|  | **Yes** | 285,331 | 41,196 | 2,167,252.73 | 19.0084 | 1.24(1.227,1.254) |  |
| **≥ 25** | **No** | 755,837 | 88,456 | 5,891,243.03 | 15.0148 | 1(Ref.) |  |
|  | **Yes** | 162,552 | 28,124 | 1,227,160.54 | 22.9179 | 1.214(1.198,1.23) |  |
| **DM** |  |  |  |  |  |  |  |
| **No** | **No** | 1,944,483 | 186,697 | 15,310,778.86 | 12.1938 | 1(Ref.) | <.0001 |
|  | **Yes** | 394,122 | 55,459 | 3,022,535.82 | 18.3485 | 1.249(1.237,1.261) |  |
| **Yes** | **No** | 238,649 | 45,676 | 1,733,006.62 | 26.3565 | 1(Ref.) |  |
|  | **Yes** | 53,761 | 13,861 | 371,877.45 | 37.273 | 1.174(1.152,1.197) |  |
| **Hypertension** |  |  |  |  |  |  |  |
| **No** | **No** | 1,456,960 | 112,828 | 11,624,668.26 | 9.7059 | 1(Ref.) | 0.0629 |
|  | **Yes** | 274,913 | 29,766 | 2,158,439.48 | 13.7905 | 1.226(1.21,1.242) |  |
| **Yes** | **No** | 726,172 | 119,545 | 5,419,117.21 | 22.0599 | 1(Ref.) |  |
|  | **Yes** | 172,970 | 39,554 | 1,235,973.79 | 32.0023 | 1.236(1.222,1.25) |  |
| **Dyslipidemia** |  |  |  |  |  |  |  |
| **No** | **No** | 1,709,357 | 161,413 | 13,431,237.64 | 12.0177 | 1(Ref.) | <.0001 |
|  | **Yes** | 330,419 | 44,857 | 2,532,042.75 | 17.7157 | 1.245(1.232,1.258) |  |
| **Yes** | **No** | 473,775 | 70,960 | 3,612,547.84 | 19.6426 | 1(Ref.) |  |
|  | **Yes** | 117,464 | 24,463 | 862,370.52 | 28.3672 | 1.206(1.188,1.223) |  |
| **Smoke** |  |  |  |  |  |  |  |
| **Non** | **No** | 1,343,351 | 160,916 | 10,464,765.88 | 15.3769 | 1(Ref.) | 0.0005 |
|  | **Yes** | 313,555 | 50,673 | 2,381,219.1 | 21.2803 | 1.219(1.207,1.231) |  |
| **Past** | **No** | 344,539 | 34,166 | 2,691,722.1 | 12.693 | 1(Ref.) |  |
|  | **Yes** | 67,853 | 10,224 | 510,609.76 | 20.0231 | 1.241(1.213,1.269) |  |
| **Current** | **No** | 495,242 | 37,291 | 3,887,297.49 | 9.593 | 1(Ref.) |  |
|  | **Yes** | 66,475 | 8,423 | 502,584.41 | 16.7594 | 1.283(1.253,1.315) |  |
| **CKD** |  |  |  |  |  |  |  |
| **No** | **No** | 2,019,395 | 203,412 | 15,843,110.79 | 12.8391 | 1(Ref.) | 0.1178 |
|  | **Yes** | 408,349 | 59,471 | 3,120,755.91 | 19.0566 | 1.23(1.219,1.241) |  |
| **Yes** | **No** | 163,737 | 28,961 | 1,200,674.68 | 24.1206 | 1(Ref.) |  |
|  | **Yes** | 39,534 | 9,849 | 273,657.36 | 35.9903 | 1.231(1.203,1.26) |  |
| **Drink** |  |  |  |  |  |  |  |
| **Non** | **No** | 1,227,556 | 155,734 | 9,474,767.84 | 16.4367 | 1(Ref.) | 0.0092 |
|  | **Yes** | 292,134 | 50,821 | 2,185,228.64 | 23.2566 | 1.225(1.212,1.237) |  |
| **Mild** | **No** | 782,840 | 61,550 | 6,218,300.7 | 9.8982 | 1(Ref.) |  |
|  | **Yes** | 133,178 | 15,659 | 1,036,541.13 | 15.107 | 1.25(1.228,1.272) |  |
| **Heavy** | **No** | 172,736 | 15,089 | 1,350,716.94 | 11.1711 | 1(Ref.) |  |
|  | **Yes** | 22,571 | 2,840 | 172,643.5 | 16.4501 | 1.204(1.156,1.253) |  |
| **Exercise** |  |  |  |  |  |  |  |
| **No** | **No** | 1,745,049 | 184,357 | 13,614,238.1 | 13.5415 | 1(Ref.) | 0.8716 |
|  | **Yes** | 354,668 | 54,524 | 2,685,055.34 | 20.3065 | 1.232(1.221,1.244) |  |
| **Yes** | **No** | 438,083 | 48,016 | 3,429,547.38 | 14.0007 | 1(Ref.) |  |
|  | **Yes** | 93,215 | 14,796 | 709,357.93 | 20.8583 | 1.22(1.198,1.243) |  |
| **Low income** |  |  |  |  |  |  |  |
| **No** | **No** | 1,712,504 | 181,730 | 13,372,634.72 | 13.5897 | 1(Ref.) | 0.0068 |
|  | **Yes** | 348,068 | 53,819 | 2,637,393.75 | 20.4061 | 1.222(1.211,1.234) |  |
| **Yes** | **No** | 470,628 | 50,643 | 3,671,150.75 | 13.7949 | 1(Ref.) |  |
|  | **Yes** | 99,815 | 15,501 | 757,019.53 | 20.4764 | 1.262(1.239,1.285) |  |
| **RVO** |  |  |  |  |  |  |  |
| **No** | **No** | 2,181,556 | 231,864 | 17,033,294.75 | 13.6124 | 1(Ref.) | 0.0455 |
|  | **Yes** | 447,412 | 69,148 | 3,391,392.73 | 20.3893 | 1.231(1.22,1.241) |  |
| **Yes** | **No** | 1,576 | 509 | 10,490.72 | 48.5191 | 1(Ref.) |  |
|  | **Yes** | 471 | 172 | 3,020.54 | 56.9434 | 1.078(0.905,1.283) |  |
| **Glaucoma** |  |  |  |  |  |  |  |
| **No** | **No** | 2,156,471 | 225,197 | 16,858,327.3 | 13.3582 | 1(Ref.) | <.0001 |
|  | **Yes** | 437,809 | 66,147 | 3,327,218.2 | 19.8806 | 1.237(1.226,1.248) |  |
| **Yes** | **No** | 26,661 | 7,176 | 185,458.18 | 38.6934 | 1(Ref.) |  |
|  | **Yes** | 10,074 | 3,173 | 67,195.07 | 47.2207 | 1.097(1.052,1.144) |  |

*Follow-up duration is in person-years. †Incidence rate is presented as 1000 person-years.

Model 4 is adjusted for age, sex, smoking, drinking, regular exercise, low income, DM, hypertension, dyslipidemia, CKD, and BMI.

eTable 2. Association between cataract and Asthma subjects according to subgroup

| **Subgroup** | **Asthma** | **N** | **Cataract** | **Follow-up duration*** | **Incidence rate†** | **Model 4** | **P for interaction** |
| --- | --- | --- | --- | --- | --- | --- | --- |
| **Sex, Age** |  |  |  |  |  |  |  |
| **Male, Age < 50** | **No** | 557,275 | 14,786 | 4,562,011.75 | 3.2411 | 1(Ref.) | <.0001 |
|  | **Yes** | 7,648 | 283 | 62,407.19 | 4.5347 | 1.359(1.208,1.528) |  |
| **Male, Age ≥ 50** | **No** | 740,463 | 109,317 | 5,543,078.01 | 19.7214 | 1(Ref.) |  |
|  | **Yes** | 29,545 | 7,105 | 196,277.52 | 36.1987 | 1.291(1.26,1.322) |  |
| **Female, Age < 50** | **No** | 480,551 | 11,258 | 3,966,047.17 | 2.8386 | 1(Ref.) |  |
|  | **Yes** | 12,443 | 377 | 102,536.14 | 3.6768 | 1.224(1.105,1.357) |  |
| **Female, Age ≥ 50** | **No** | 761,053 | 146,903 | 5,712,399.59 | 25.7165 | 1(Ref.) |  |
|  | **Yes** | 42,037 | 11,664 | 293,441.38 | 39.749 | 1.203(1.18,1.226) |  |
| **Sex** |  |  |  |  |  |  |  |
| **Male** | **No** | 1,297,738 | 124,103 | 10,105,089.76 | 12.2812 | 1(Ref.) | <.0001 |
|  | **Yes** | 37,193 | 7,388 | 258,684.72 | 28.5599 | 1.282(1.252,1.313) |  |
| **Female** | **No** | 1,241,604 | 158,161 | 9,678,446.75 | 16.3416 | 1(Ref.) |  |
|  | **Yes** | 54,480 | 12,041 | 395,977.52 | 30.4083 | 1.195(1.173,1.217) |  |
| **BMI** |  |  |  |  |  |  |  |
| **< 25** | **No** | 1,657,462 | 174,072 | 12,927,954.05 | 13.4648 | 1(Ref.) | 0.0021 |
|  | **Yes** | 55,164 | 11,041 | 391,841.12 | 28.1772 | 1.256(1.232,1.281) |  |
| **≥ 25** | **No** | 881,880 | 108,192 | 6,855,582.46 | 15.7816 | 1(Ref.) |  |
|  | **Yes** | 36,509 | 8,388 | 262,821.12 | 31.9152 | 1.191(1.165,1.218) |  |
| **DM** |  |  |  |  |  |  |  |
| **No** | **No** | 2,260,444 | 226,697 | 17,766,655.75 | 12.7597 | 1(Ref.) | <.0001 |
|  | **Yes** | 78,161 | 15,459 | 566,658.93 | 27.281 | 1.257(1.236,1.278) |  |
| **Yes** | **No** | 278,898 | 55,567 | 2,016,880.76 | 27.551 | 1(Ref.) |  |
|  | **Yes** | 13,512 | 3,970 | 88,003.31 | 45.1119 | 1.147(1.11,1.185) |  |
| **Hypertension** |  |  |  |  |  |  |  |
| **No** | **No** | 1,683,654 | 135,148 | 13,420,230.94 | 10.0705 | 1(Ref.) | <.0001 |
|  | **Yes** | 48,219 | 7,446 | 362,876.8 | 20.5194 | 1.254(1.225,1.284) |  |
| **Yes** | **No** | 855,688 | 147,116 | 6,363,305.58 | 23.1194 | 1(Ref.) |  |
|  | **Yes** | 43,454 | 11,983 | 291,785.43 | 41.0678 | 1.223(1.2,1.246) |  |
| **Dyslipidemia** |  |  |  |  |  |  |  |
| **No** | **No** | 1,975,167 | 193,938 | 15,497,191.42 | 12.5144 | 1(Ref.) | 0.0048 |
|  | **Yes** | 64,609 | 12,332 | 466,088.97 | 26.4585 | 1.237(1.214,1.259) |  |
| **Yes** | **No** | 564,175 | 88,326 | 4,286,345.1 | 20.6064 | 1(Ref.) |  |
|  | **Yes** | 27,064 | 7,097 | 188,573.26 | 37.6352 | 1.217(1.188,1.247) |  |
| **Smoke** |  |  |  |  |  |  |  |
| **Non** | **No** | 1,592,541 | 197,525 | 12,382,626.01 | 15.9518 | 1(Ref.) | <.0001 |
|  | **Yes** | 64,365 | 14,064 | 463,358.97 | 30.3523 | 1.203(1.182,1.224) |  |
| **Past** | **No** | 399,227 | 41,690 | 3,110,116.57 | 13.4046 | 1(Ref.) |  |
|  | **Yes** | 13,165 | 2,700 | 92,215.3 | 29.2793 | 1.222(1.174,1.271) |  |
| **Current** | **No** | 547,574 | 43,049 | 4,290,793.93 | 10.0329 | 1(Ref.) |  |
|  | **Yes** | 14,143 | 2,665 | 99,087.96 | 26.8953 | 1.373(1.319,1.429) |  |
| **CKD** |  |  |  |  |  |  |  |
| **No** | **No** | 2,346,091 | 246,508 | 18,372,952.47 | 13.4169 | 1(Ref.) | <.0001 |
|  | **Yes** | 81,653 | 16,375 | 590,914.23 | 27.7113 | 1.232(1.212,1.252) |  |
| **Yes** | **No** | 193,251 | 35,756 | 1,410,584.04 | 25.3484 | 1(Ref.) |  |
|  | **Yes** | 10,020 | 3,054 | 63,748 | 47.9074 | 1.217(1.173,1.263) |  |
| **Drink** |  |  |  |  |  |  |  |
| **Non** | **No** | 1,455,081 | 191,659 | 11,205,828.52 | 17.1035 | 1(Ref.) | 0.0003 |
|  | **Yes** | 64,609 | 14,896 | 454,167.97 | 32.7984 | 1.219(1.198,1.239) |  |
| **Mild** | **No** | 893,200 | 73,408 | 7,084,963.29 | 10.3611 | 1(Ref.) |  |
|  | **Yes** | 22,818 | 3,801 | 169,878.53 | 22.3748 | 1.264(1.223,1.307) |  |
| **Heavy** | **No** | 191,061 | 17,197 | 1,492,744.7 | 11.5204 | 1(Ref.) |  |
|  | **Yes** | 4,246 | 732 | 30,615.73 | 23.9093 | 1.248(1.159,1.345) |  |
| **Exercise** |  |  |  |  |  |  |  |
| **No** | **No** | 2,025,884 | 223,222 | 15,774,242.58 | 14.151 | 1(Ref.) | 0.0309 |
|  | **Yes** | 73,833 | 15,659 | 525,050.86 | 29.8238 | 1.224(1.204,1.244) |  |
| **Yes** | **No** | 513,458 | 59,042 | 4,009,293.94 | 14.7263 | 1(Ref.) |  |
|  | **Yes** | 17,840 | 3,770 | 129,611.37 | 29.087 | 1.245(1.204,1.286) |  |
| **Low income** |  |  |  |  |  |  |  |
| **No** | **No** | 1,989,757 | 220,307 | 15,506,207.67 | 14.2077 | 1(Ref.) | 0.4081 |
|  | **Yes** | 70,815 | 15,242 | 503,820.8 | 30.2528 | 1.232(1.212,1.252) |  |
| **Yes** | **No** | 549,585 | 61,957 | 4,277,328.84 | 14.485 | 1(Ref.) |  |
|  | **Yes** | 20,858 | 4,187 | 150,841.44 | 27.7576 | 1.214(1.176,1.253) |  |
| **RVO** |  |  |  |  |  |  |  |
| **No** | **No** | 2,537,419 | 281,638 | 19,770,736.93 | 14.2452 | 1(Ref.) | 0.1251 |
|  | **Yes** | 91,549 | 19,374 | 653,950.55 | 29.6261 | 1.228(1.21,1.246) |  |
| **Yes** | **No** | 1,923 | 626 | 12,799.58 | 48.9079 | 1(Ref.) |  |
|  | **Yes** | 124 | 55 | 711.68 | 77.2814 | 1.199(0.907,1.587) |  |
| **Glaucoma** |  |  |  |  |  |  |  |
| **No** | **No** | 2,505,027 | 272,765 | 19,546,204.16 | 13.9549 | 1(Ref.) | <.0001 |
|  | **Yes** | 89,253 | 18,579 | 639,341.34 | 29.0596 | 1.243(1.224,1.262) |  |
| **Yes** | **No** | 34,315 | 9,499 | 237,332.35 | 40.024 | 1(Ref.) |  |
|  | **Yes** | 2,420 | 850 | 15,320.9 | 55.4798 | 1(0.932,1.074) |  |

*Follow-up duration is in person-years. †Incidence rate is presented as 1000 person-years.

Model 4 is adjusted for age, sex, smoking, drinking, regular exercise, low income, DM, hypertension, dyslipidemia, CKD, and BMI.

eTable 3. Association between cataract and Rhinitis subjects according to subgroup

| **Subgroup** | **Rhinitis** | **N** | **Cataract** | **Follow-up duration*** | **Incidence rate†** | **Model 4** | **P for interaction** |
| --- | --- | --- | --- | --- | --- | --- | --- |
| **Sex, Age** |  |  |  |  |  |  |  |
| **Male, Age < 50** | **No** | 504,350 | 13,239 | 4,127,934.23 | 3.2072 | 1(Ref.) | 0.0044 |
|  | **Yes** | 60,573 | 1,830 | 496,484.71 | 3.6859 | 1.173(1.117,1.232) |  |
| **Male, Age ≥ 50** | **No** | 663,845 | 95,380 | 4,971,564.99 | 19.1851 | 1(Ref.) |  |
|  | **Yes** | 106,163 | 21,042 | 767,790.54 | 27.4059 | 1.239(1.221,1.258) |  |
| **Female, Age < 50** | **No** | 409,039 | 9,378 | 3,375,998.58 | 2.7778 | 1(Ref.) |  |
|  | **Yes** | 83,955 | 2,257 | 692,584.73 | 3.2588 | 1.172(1.12,1.228) |  |
| **Female, Age ≥ 50** | **No** | 653,744 | 124,677 | 4,905,522.02 | 25.4156 | 1(Ref.) |  |
|  | **Yes** | 149,346 | 33,890 | 1,100,318.95 | 30.8002 | 1.215(1.201,1.23) |  |
| **Sex** |  |  |  |  |  |  |  |
| **Male** | **No** | 1,168,195 | 108,619 | 9,099,499.23 | 11.9368 | 1(Ref.) | 0.5371 |
|  | **Yes** | 166,736 | 22,872 | 1,264,275.25 | 18.091 | 1.231(1.213,1.249) |  |
| **Female** | **No** | 1,062,783 | 134,055 | 8,281,520.6 | 16.1872 | 1(Ref.) |  |
|  | **Yes** | 233,301 | 36,147 | 1,792,903.67 | 20.1612 | 1.221(1.207,1.235) |  |
| **BMI** |  |  |  |  |  |  |  |
| **< 25** | **No** | 1,456,153 | 149,773 | 11,354,224.52 | 13.1909 | 1(Ref.) | 0.1326 |
|  | **Yes** | 256,473 | 35,340 | 1,965,570.65 | 17.9795 | 1.232(1.218,1.246) |  |
| **≥ 25** | **No** | 774,825 | 92,901 | 6,026,795.3 | 15.4147 | 1(Ref.) |  |
|  | **Yes** | 143,564 | 23,679 | 1,091,608.27 | 21.6918 | 1.211(1.194,1.228) |  |
| **DM** |  |  |  |  |  |  |  |
| **No** | **No** | 1,984,851 | 194,864 | 15,599,447.01 | 12.4917 | 1(Ref.) | <.0001 |
|  | **Yes** | 353,754 | 47,292 | 2,733,867.66 | 17.2986 | 1.241(1.229,1.254) |  |
| **Yes** | **No** | 246,127 | 47,810 | 1,781,572.81 | 26.8358 | 1(Ref.) |  |
|  | **Yes** | 46,283 | 11,727 | 323,311.26 | 36.2715 | 1.174(1.151,1.198) |  |
| **Hypertension** |  |  |  |  |  |  |  |
| **No** | **No** | 1,481,341 | 116,736 | 11,805,944.48 | 9.8879 | 1(Ref.) | 0.6112 |
|  | **Yes** | 250,532 | 25,858 | 1,977,163.26 | 13.0783 | 1.216(1.2,1.232) |  |
| **Yes** | **No** | 749,637 | 125,938 | 5,575,075.34 | 22.5895 | 1(Ref.) |  |
|  | **Yes** | 149,505 | 33,161 | 1,080,015.66 | 30.7042 | 1.231(1.216,1.246) |  |
| **Dyslipidemia** |  |  |  |  |  |  |  |
| **No** | **No** | 1,743,217 | 168,067 | 13,671,697.05 | 12.2931 | 1(Ref.) | 0.0002 |
|  | **Yes** | 296,559 | 38,203 | 2,291,583.33 | 16.671 | 1.241(1.227,1.254) |  |
| **Yes** | **No** | 487,761 | 74,607 | 3,709,322.77 | 20.1134 | 1(Ref.) |  |
|  | **Yes** | 103,478 | 20,816 | 765,595.59 | 27.1893 | 1.197(1.179,1.216) |  |
| **Smoke** |  |  |  |  |  |  |  |
| **Non** | **No** | 1,375,810 | 168,207 | 10,695,190.36 | 15.7273 | 1(Ref.) | 0.0997 |
|  | **Yes** | 281,096 | 43,382 | 2,150,794.62 | 20.1702 | 1.218(1.205,1.231) |  |
| **Past** | **No** | 351,498 | 35,620 | 2,739,724.59 | 13.0013 | 1(Ref.) |  |
|  | **Yes** | 60,894 | 8,770 | 462,607.27 | 18.9578 | 1.233(1.205,1.263) |  |
| **Current** | **No** | 503,670 | 38,847 | 3,946,104.87 | 9.8444 | 1(Ref.) |  |
|  | **Yes** | 58,047 | 6,867 | 443,777.02 | 15.474 | 1.256(1.224,1.289) |  |
| **CKD** |  |  |  |  |  |  |  |
| **No** | **No** | 2,061,765 | 212,074 | 16,145,930.95 | 13.1348 | 1(Ref.) | 0.9257 |
|  | **Yes** | 365,979 | 50,809 | 2,817,935.75 | 18.0306 | 1.224(1.212,1.236) |  |
| **Yes** | **No** | 169,213 | 30,600 | 1,235,088.87 | 24.7755 | 1(Ref.) |  |
|  | **Yes** | 34,058 | 8,210 | 239,243.17 | 34.3165 | 1.226(1.196,1.256) |  |
| **Drink** |  |  |  |  |  |  |  |
| **Non** | **No** | 1,260,722 | 163,542 | 9,704,215.19 | 16.8527 | 1(Ref.) | 0.229 |
|  | **Yes** | 258,968 | 43,013 | 1,955,781.29 | 21.9927 | 1.22(1.207,1.233) |  |
| **Mild** | **No** | 794,964 | 63,614 | 6,307,782.76 | 10.085 | 1(Ref.) |  |
|  | **Yes** | 121,054 | 13,595 | 947,059.06 | 14.355 | 1.237(1.214,1.26) |  |
| **Heavy** | **No** | 175,292 | 15,518 | 1,369,021.87 | 11.3351 | 1(Ref.) |  |
|  | **Yes** | 20,015 | 2,411 | 154,338.57 | 15.6215 | 1.21(1.159,1.263) |  |
| **Exercise** |  |  |  |  |  |  |  |
| **No** | **No** | 1,783,919 | 192,750 | 13,886,579.02 | 13.8803 | 1(Ref.) | 0.3223 |
|  | **Yes** | 315,798 | 46,131 | 2,412,714.41 | 19.12 | 1.228(1.216,1.241) |  |
| **Yes** | **No** | 447,059 | 49,924 | 3,494,440.8 | 14.2867 | 1(Ref.) |  |
|  | **Yes** | 84,239 | 12,888 | 644,464.51 | 19.998 | 1.209(1.186,1.233) |  |
| **Low income** |  |  |  |  |  |  |  |
| **No** | **No** | 1,749,681 | 189,837 | 13,633,751.5 | 13.924 | 1(Ref.) | 0.0039 |
|  | **Yes** | 310,891 | 45,712 | 2,376,276.97 | 19.2368 | 1.216(1.203,1.228) |  |
| **Yes** | **No** | 481,297 | 52,837 | 3,747,268.33 | 14.1001 | 1(Ref.) |  |
|  | **Yes** | 89,146 | 13,307 | 680,901.95 | 19.5432 | 1.26(1.236,1.284) |  |
| **RVO** |  |  |  |  |  |  |  |
| **No** | **No** | 2,229,340 | 242,139 | 17,370,166.44 | 13.9399 | 1(Ref.) | 0.1095 |
|  | **Yes** | 399,628 | 58,873 | 3,054,521.04 | 19.2741 | 1.225(1.214,1.236) |  |
| **Yes** | **No** | 1,638 | 535 | 10,853.38 | 49.2934 | 1(Ref.) |  |
|  | **Yes** | 409 | 146 | 2,657.88 | 54.931 | 1.069(0.889,1.285) |  |
| **Glaucoma** |  |  |  |  |  |  |  |
| **No** | **No** | 2,203,167 | 235,094 | 17,188,456.18 | 13.6774 | 1(Ref.) | <.0001 |
|  | **Yes** | 391,113 | 56,250 | 2,997,089.32 | 18.7682 | 1.23(1.219,1.242) |  |
| **Yes** | **No** | 27,811 | 7,580 | 192,563.65 | 39.3636 | 1(Ref.) |  |
|  | **Yes** | 8,924 | 2,769 | 60,089.6 | 46.0812 | 1.114(1.066,1.163) |  |

*Follow-up duration is in person-years. †Incidence rate is presented as 1000 person-years.

Model 4 is adjusted for age, sex, smoking, drinking, regular exercise, low income, DM, hypertension, dyslipidemia, CKD, and BMI.
